# Environmental endocrine disruptors and endometrial cancer risk: a systematic review and meta-analysis of cadmium and polychlorinated biphenyls, with emerging evidence on PFAS, phthalates, and bisphenols

**DOI:** 10.3389/fonc.2026.1848363

**Published:** 2026-05-28

**Authors:** Lutian Gong, Wenping Lu, Yujing Zhao, Heting Mei, Xiangyang Zhang

**Affiliations:** 1 Department of Oncology, China Academy of Chinese Medical Sciences Guang’anmen Hospital, Beijing, China; 2 Beijing University of Chinese Medicine, Beijing, China

**Keywords:** cadmium, endometrial cancer, environmental endocrine-disrupting chemicals, meta-analysis, PCBs, systematic review

## Abstract

**Background:**

Environmental endocrine-disrupting chemicals (EDCs) have been implicated in hormone-related carcinogenesis, however, their associations with endometrial cancer risk remain unclear; therefore, this study aimed to systematically evaluate the relationship between multiple classes of EDCs and the endometrial cancer risk.

**Methods:**

The PubMed, Web of Science, EMBASE, and Cochrane Library databases were searched for studies published up to November 30, 2025. Studies evaluating the association between EDCs exposure and endometrial cancer risk were included. When sufficient data were available, meta-analyses of cadmium and polychlorinated biphenyls (PCBs) were conducted using random-effects models, with subgroup and sensitivity analyses performed. Evidence on per- and polyfluoroalkyl substances (PFAS), phthalates, and bisphenols was summarized through a structured narrative review. All statistical analyses were performed using Stata 18.0.

**Results:**

A total of 14 studies were included. The meta-analysis showed that higher cadmium exposure was associated with an increased risk of endometrial cancer (RR = 1.22, 95% CI: 1.01-1.47), with considerable heterogeneity across studies (I²= 72.17%). Subgroup analyses suggested that the association was not statistically significant for dietary cadmium exposure or among women undergoing hormone therapy, and a marginally positive association was observed in postmenopausal women (RR = 1.27, 95% CI: 0.99-1.64). No significant association was identified between PCBs exposure and endometrial cancer risk (RR = 1.11, 95% CI: 0.79-1.56), and heterogeneity was low (I² = 0.0%). Findings from case-control studies and subgroup analyses stratified by PCBs functional classification (Estrogenic and Enzyme-inducing types) were consistent, with no significant associations detected. Owing to the limited number of available studies, quantitative synthesis was not feasible for PFAS, phthalates, or bisphenols.

**Conclusion:**

Current evidence suggests a potential link between cadmium exposure and endometrial cancer, though substantial heterogeneity warrants caution. No consistent association was observed for PCBs. Evidence on emerging EDCs, including PFAS, phthalates, and bisphenols, remains limited and exploratory. Well-designed prospective studies with improved exposure assessment and standardized confounder control are needed.

## 1 Introduction

Endometrial cancer is a common gynecologic malignancy with a steadily increasing incidence. Its pathogenesis is multifactorial, involving established risk factors such as obesity, metabolic syndrome, and hormonal exposure, as well as environmental influences (1, 2). With ongoing industrialization and lifestyle changes, both the diversity and levels of chemical exposures in the population have increased, bringing environmental endocrine-disrupting chemicals (EDCs) to the forefront of gynecologic cancer research (3). EDCs can interfere with hormone synthesis, secretion, transport, and metabolism, thereby altering estrogen signaling pathways (4). Given the high sensitivity of endometrial tissue to hormonal fluctuations, EDCs exposure may be implicated in the development of endometrial cancer (5).

EDCs originate from diverse sources, including industrial emissions, agricultural activities, plastic products, persistent organic pollutants, and heavy metals (6). Cadmium, a heavy metal characterized by long-term environmental and biological accumulation, is considered as a potential carcinogen with estrogen-mimicking properties (7). Polychlorinated biphenyls (PCBs), as representative persistent organic pollutants, may also contribute to tumorigenesis by modulating estrogen receptors, oxidative stress, and immune regulation (8). In addition, increasing attention has been directed toward emerging EDCs, such as per- and polyfluoroalkyl substances (PFAS), phthalates, and bisphenols, which are frequently detected in human urine and blood samples. These compounds may increase cancer risk by disrupting estrogen metabolism and influencing endometrial proliferation and inflammation (9–11). Although studies examining the relationship between EDC exposure and endometrial cancer have grown in number, findings remain inconsistent. Variations in exposure assessment methods, study designs, sample sizes, and statistical modeling approaches across studies limit the overall comparability and strength of the evidence (12, 13).

In this context, systematically synthesizing the available epidemiological evidence is essential to clarify the potential associations between EDCs and endometrial cancer risk. This study conducted a systematic review and meta-analysis to quantitatively evaluate the associations of cadmium and PCBs exposure with endometrial cancer risk. For emerging EDCs, including PFAS, phthalates, and bisphenols, which were not suitable for meta-analysis, a structured narrative review was performed. This approach was designed to assess the strength and uncertainty of the evidence across different EDCs and to inform future research and environmental health decision-making.

## 2 Materials and methods

This systematic review and meta-analysis was conducted in accordance with the PRISMA guidelines (14). The study was registered in PROSPERO (CRD420251241318).

### 2.1 Data sources

We searched PubMed, EMBASE, Web of Science, and the Cochrane Library for studies published up to November 30, 2025, on EDCs and endometrial cancer (see **Supplementary File 1** for details). Reference lists were also screened. Although Scopus was not searched, these sources were deemed sufficient to comprehensively capture relevant studies.

### 2.2 Study selection

The titles and abstracts of retrieved records were independently screened by two investigators (GLT and ZYJ) according to the predefined inclusion and exclusion criteria:

#### 2.2.1 Inclusion criteria

(1) The study population consisted of humans, with clearly reported endometrial cancer incidence or incidence or diagnosis (2) The study assessed exposure to five types of EDCs: cadmium, PCBs, PFAS, phthalates, and bisphenols; (3) The study design was a cohort, case-control, or nested case-control study; (4) Exposure assessment was based on biological measurements (e.g., serum, plasma, or urine) or clearly defined environmental/occupational exposures; (5) Studies reported effect estimates for endometrial cancer risk, including odds ratios (ORs), relative risks (RRs), incidence rate ratios (IRRs), or hazard ratios (HRs), together with 95% confidence intervals or sufficient data to derive them. Alternatively, studies were eligible if they provided sufficient data for quantitative synthesis (e.g., mean ± SD) or at least reported statistical comparisons between exposure groups.

#### 2.2.2 Exclusion criteria

(1) Animal studies, *in vitro* experiments, mechanistic studies, reviews or meta-analyses, conference abstracts, case reports, and commentary articles; (2) Studies that did not report endometrial cancer outcomes separately, or combined them with other cancers without extractable data; (3) Studies reporting only mixed exposures without distinguishing individual chemicals; (4) Duplicate publications, retaining only the study with the largest sample size or most complete information.

### 2.3 Data extraction and quality assessment

Data extraction was independently performed by two investigators (GLT and MHT). Any discrepancies were resolved through discussion, and when consensus could not be reached, a third reviewer (LWP) made the final decision. The following information was extracted: first author, year of publication, study country or region, and study design. Additional variables included menopausal status, type of biological specimen, and exposure category. Sample size and effect estimates for endometrial cancer risk with corresponding 95% confidence intervals were also collected. When multiple analytical models were reported within a study, estimates from the multivariable-adjusted model were preferentially selected to minimize confounding bias. For the meta-analysis, risk estimates comparing the highest exposure category with the lowest exposure category were preferentially used. Major confounding factors adjusted for in the original studies, such as age, body mass index (BMI), and hormone use, were also documented.

The methodological quality of the included studies was independently evaluated by two investigators (GLT and ZXY). Both case-control and cohort studies were assessed using the Newcastle-Ottawa Scale (15). The scale evaluates study quality across three domains: selection of study participants, comparability between groups, and assessment of exposure or outcome. The total score ranges from 0 to 9 points. Studies with scores ≥7 were considered high quality, those scoring 5–6 were regarded as moderate quality, and studies with scores <5 were classified as low quality.

### 2.4 Data analysis

When at least three studies reported comparable risk estimates for the same exposure, a meta-analysis was conducted to evaluate the associations of cadmium and PCBs with endometrial cancer risk. The pooled effect measures included ORs, HRs, RRs, and IRRs. Given the relatively low incidence of endometrial cancer, these measures (ORs, HRs, IRRs) were treated as approximate relative risks under the rare disease assumption (16). For PFAS, phthalates, and bisphenols, the number of studies and heterogeneity in exposure measurement were insufficient for meta-analysis, thus a narrative summary was performed. To ensure comparability across studies, all effect estimates were logarithmically transformed and expressed as the natural logarithm of the relative risk (lnRR). Standard errors were calculated using the formula: ES=(lnuci-lnlci)/(2 × 1.96). Meta-analyses were performed using Stata 18.0. A random-effects model based on the restricted maximum likelihood (REML) method was applied to pool the effect estimates. Between-study heterogeneity was assessed using the I² statistic (17). To further explore potential sources of heterogeneity, the following study-level covariates were considered when sufficient data were available: exposure assessment method (diet-based vs. biomarker-based), study design (cohort vs. case-control), and menopausal status (postmenopausal vs. mixed populations). When heterogeneity was not substantial (I²< 50% and p > 0.1), results from the fixed-effects model were additionally reported as part of sensitivity analyses to examine the robustness of the findings. Publication bias was not evaluated due to the limited number of included studies.

## 3 Results

### 3.1 Search results and study characteristics

A total of 6,900 records were identified through the literature search. After removing duplicates, 1,977 records remained for further screening. Following the review of titles, abstracts, and extractable information, 96 articles were retained for full-text assessment. After full-text review, 82 studies were excluded. The reasons for exclusion included 60 cross-sectional studies, 9 studies that did not report ORs, HRs, RRs, or 95% confidence intervals, and 13 studies involving endocrine-disrupting chemicals outside the predefined scope. Ultimately, 14 studies were included in the final analysis.

**Figure 1:**
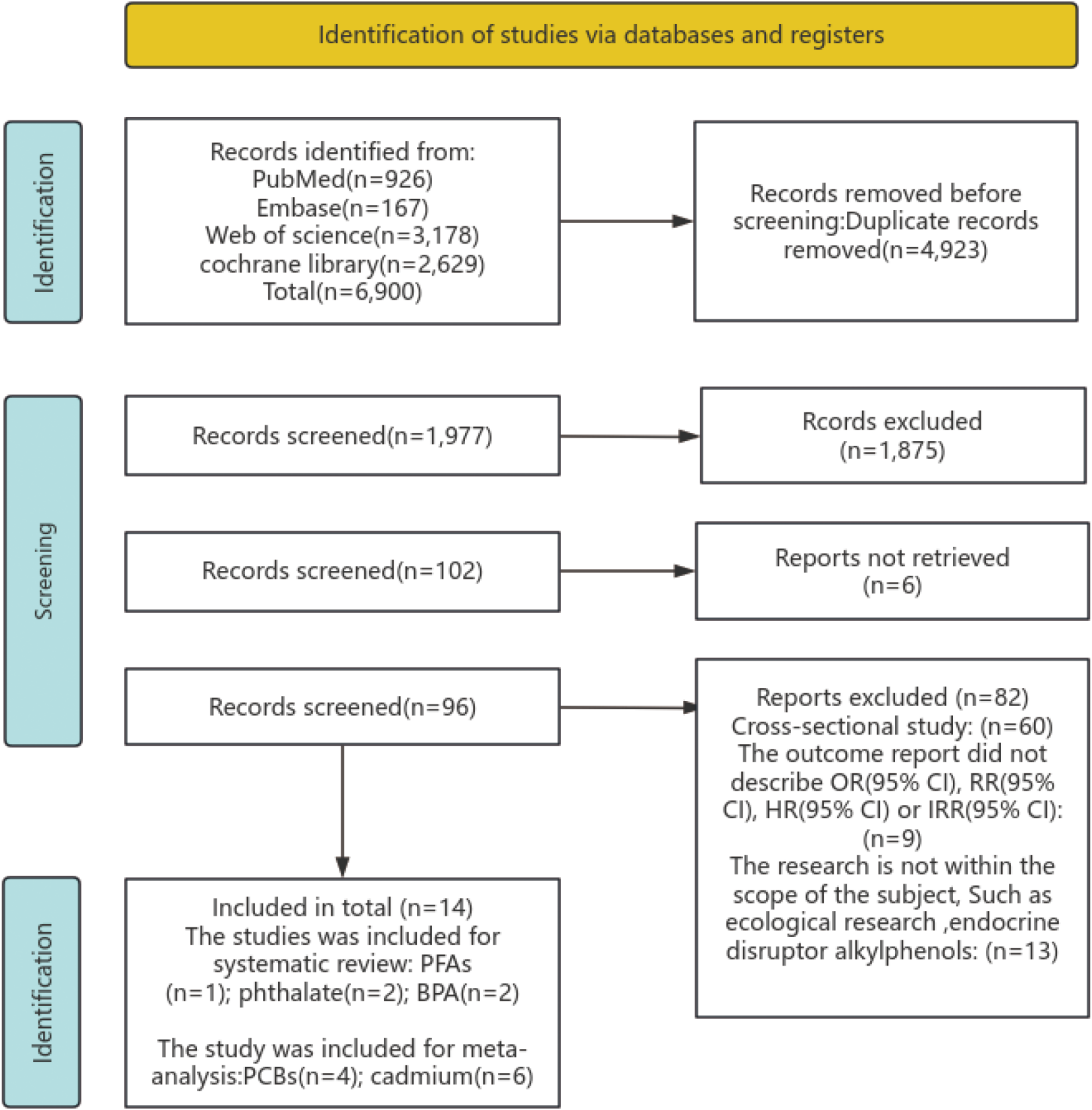
Flow diagram of the literature screening process according to the PRISMA guidelines.

Among the included studies, only one study examined the association between PFAS and endometrial cancer (18). Two studies investigated phthalates (10, 19) and two studies investigated bisphenols (10, 20), with one study (Danja Sarink) assessing both classes (10). Four studies assessed the relationship between PCBs and endometrial cancer (13, 21–23). Six studies examined the association between cadmium exposure and endometrial cancer (24–29). The literature screening process is presented in **Figure 1**, and the main characteristics of the included studies are summarized in **Table 1**.

**Table 1:** Characteristics of included studies.

Number	Author,year	Country	Study design	Menopausal status	Sample tested	Exposure_type	Case/control	Effect	Effect estimate (95% CI)	Adjusted_Covariates	Main conclusion
Meta-analysis											
PCBs											
1	Weiderpass et al., 2000 (22)	Sweden	Case-control	Not mentioned	serum	PCB105, PCB118, PCB138, PCB153, PCB156, PCB167, PCB180.	154/205	OR	Total PCBs: 1.2 (0.6-2.2) PCB105: 0.8 (0.4-1.6) PCB118: 1.4 (0.7-2.8) PCB138:0.9 (0.5-1.7) PCB153:0.9 (0.5-1.7) PCB156: 1.0 (0.5-2.0) PCB167: 1.9 (0.9-3.9) PCB180:1.2 (0.6-2.2)	Age, body mass index (BMI)	No significant association with endometrial cancer was observed.
2	Sturgeon et al., 1998 (23)	USA	Case-control	Not mentioned	serum	Estrogenic PCBs, Anti-estrogenic PCBs, Enzyme-inducing PCBs.	90/90	RR	Total PCBs:0.9 (0.4-2.5) Estrogenic PCBs:1.3 (0.5-3.2) Anti-estrogenic PCBs:1.1 (0.4-3.1) Enzyme-inducing PCBs:0.6 (0.2-1.6)	cigarette smoking, diet, and weight	No significant association with endometrial cancer was observed.
3	Hardell et al., 2004 (13)	Sweden	Case-control	Among cases, 72 were postmenopausal, compared with 30 in controls.	adipose tissue	Estrogenic PCBs, Enzyme-inducing PCBs.	76/39	OR	Total PCBs:0.9(0.4-2.3) Estrogenic PCBs:1.0(0.4-2.4) Enzyme-inducing PCBs:1.1(0.5-2.6)	Age, BMI	No significant association with endometrial cancer was observed.
4	Donat-Vargas et al., 2016 (21)	Sweden	cohort	Not mentioned	Dietary PCB exposure	PCBs	437/36777	RR	Total PCBs:1.21 (0.73, 2.01)	Age, postsecondary education, family history of breast cancer, oophorectomy, history of diabetes, BMI, weight loss>5 kg within 1 year, age at menarche ≤12 years, use of oral contraceptives, parity, age at first birth ≥30 years, age at menopause≥51 years, ever use of postmenopausal hormones, smoking habits, leisure-time inactivity, time spent walking or bicycling, alcohol consumption and total energy intake,dietary EPA-DHA intake.	No significant association with endometrial cancer was observed.
Cadmium											
5	McElroy et al., 2017 (24)	USA	Case-control	Participants were aged 18–81 years.	urine	Cadmium	631/879	OR	1.22 (1.03, 1.44) postmenopausal women:1.25(1.04-1.50) HRT users:0.67(0.53-0.84)	Urine concentration of creatinine, BMI	Cadmium exposure was associated with an increased risk of endometrial cancer.
6	Michalczyk et al., 2022 (29)	Poland	Case-control	Not mentioned	serum	Cadmium, Cu, Zn, Pb, Co	21/89	OR	1.49(1.31-1.63) postmenopausal women:1.89(1.36-1.94)	Age, Weight, BMI, Menopause, Smoking, Diabetes type 2	High cadmium levels are an independent risk factor for endometrial cancer.
7	Sawada et al., 2012 (27)	Japan	cohort	Participants were aged 45–74years.	dietary Cadmium exposure	Cadmium	75/52,486	HR	1.49 (0.63-3.53) premenopausal Women: 1.69 (0.42- 6.87) postmenopausal women:1.45 (0.49-4.36)	Age, area, BMI, smoking status, frequency of alcohol intake, leisure-time physical activity, intake of meat, soybean, vegetable, and fruit, menopausal status, and use of exogenous female hormones.	No evidence links cadmium intake to endometrial cancer.
8	Akesson et al.,2008 (28)	Sweden	cohort	Postmenopausal women	dietary Cadmium exposure	Cadmium	378/30,210	RR	1.39 (1.04–1.86) HRT users:1.10(0.65-1.84)	Age, postsecondary education, BMI, use of postmenopausal hormones, smoking status, parity, age at menarche, age at menopause, and leisure time physical inactivity, intake of vegetables, whole grains, and potatoes	Cadmium intake was associated with increased endometrial cancer risk, showing a positive dose–response relationship among postmenopausal women.
9	Eriksen et al,. 2014 (26)	Danish	cohort	Postmenopausal women	dietary Cadmium exposure	Cadmium	192/23,815	IRR	1.08(0.76-1.53) HRT users:1.35(0.67-2.72)	Educational level, smoking status, number of births, age at first birth, HRT status, HRT use, age at menarche, BMI, height, physical activity,alcohol intake.	No evidence links cadmium intake to endometrial cancer.
10	Adams et al., 2014 (25)	USA	cohort	Postmenopausal women	dietary Cadmium exposure	Cadmium	1,198/155,069	HR	0.86(0.67-1.11)	Total energy intake, age, study component, BMI, smoking, alcohol consumption,race/ethnicity, education, physical activity, age at first birth, age at menarche, age at menopause, unopposed estrogen use, and estrogen and progesterone use, daily vegetable servings and daily grain servings.	Cadmium intake is rarely associated with EC.
Narrative review											
PFAs											
11	Madrigal et al., 2025 (18)	USA	Case-control	Most women (∼98%) were postmenopausal	serum	PFOA, PFOS, MeFOSAA, PFHxS, PFNA, PFDA, EtFOSAA, PFUnDA	430/448	OR	PFOA:0.71 (0.50–1.00) PFOS:1.00 (0.72–1.39) MeFOSAA:0.98 (0.81–1.20) PFHxS:0.92 (0.79–1.07) PFNA:1.08 (0.79–1.46) PFDA:1.01 (0.72–1.42) EtFOSAA:1.08 (0.93–1.26) PFUnDA:1.07 (0.79–1.44)	Age, duration of oral contraceptive use, duration of menopausal hormone therapy use, smoking status, calendar year of blood draw, BMI, parity, and years since last menstrual period.	A significant nonlinear association between EtFOSA concentration and endometrial cancer was observed only in the subgroup analysis.
Phthalates and bisphenols											
12	Lin et al., 2025 (10)	China	Case-control	Not mentioned	Urine	MBzP, MECPP, MEHHP,MEHP, MEOHP, MnBP, MEP, MMP	116/116	OR	MBzp:3.712(1.464-9.414) MECPP:1.352(0.260–7.034) MEHHP:3.110(0.657–14.715) MEHP:1.147(0.366–3.597) MEOHP:1.358(0.254–7.252) MnBP:0.970(0.323–2.911) MEP:1.719(0.702–4.209) MMP:1.817(0.689–4.796)	Age, BMI, Alcohol drinking, Meals out, Regular handwashing, Plastic container use, Chilled-ready meals, Frequent seafood consumption, Urinate, Occupation, Chronic disease, Medicine, Family history of cancer, Family history of chronic disease.	Urinary MBzP levels were independently higher in women with EC than in controls.
13	Sarink et al., 2021 (19)	USA	Case-control	Postmenopausal women	Urine	Bisphenol A (BPA), Phthalate(MBzP, MECPP,MEHHP,MEHP, MEOHP, MEP, MiBP, MMP, MnBP, PA	139/139	OR	Total phthalates:1.22 (0.61, 2.43); MBzP:1.07 (0.55, 2.11) MECPP:1.52 (0.74, 3.13) MEHHP:0.95 (0.48, 1.87) MEHP:1.43 (0.75, 2.75) MEOHP:1.17 (0.57, 2.42) MEP:0.93 (0.43, 2.00) MiBP:1.85 (0.90, 3.82) MMP:0.59 (0.27, 1.31) MnBP:1.82 (0.81, 4.10) BPA:1.21(0.60-2.44)	BMI, diabetes, and Mediterranean Diet Score.	mono-*n*-butyl phthalate (MnBP) excretion was positively associated with endometrial cancer risk.
Bisphenols											
14	Aquino et al., 2019 (20)	Italy	Case-control	Postmenopausal women aged 50–80 years	blood, urine,Endometrium tissue sample	Bisphenol A (BPA)	17/7	**/**	Total BPA (ng/mL)(Blood):1.43 ± 0.26 Total BPA (ng/mL) (Urine):4.33 ± 1.29 SMD (Hedges’g) = 1.42 (0.45–2.39)	Not specified.	BPA may indirectly promote abnormal proliferation of endometrial cells.

### 3.2 Study quality assessment

Among the included studies, five were conducted in the United States and four in Sweden. According to the study design, five studies were cohort studies and nine were case-control studies. The quality scores of the included studies ranged from 4 to 9. Detailed scoring criteria are provided in **Supplementary File 2**.

### 3.3 Association of PCBs exposure with endometrial cancer risk

A total of four studies were included in the analysis, and the pooled results based on a random-effects model indicated no statistically significant association between PCBs exposure and endometrial cancer risk (RR = 1.11, 95% CI: 0.79-1.56). No heterogeneity was observed among the included studies (I² = 0.0%, p = 0.55). Among these studies, Donat-Vargas et al. contributed the greatest weight (44.48%).

**Figure 2:**
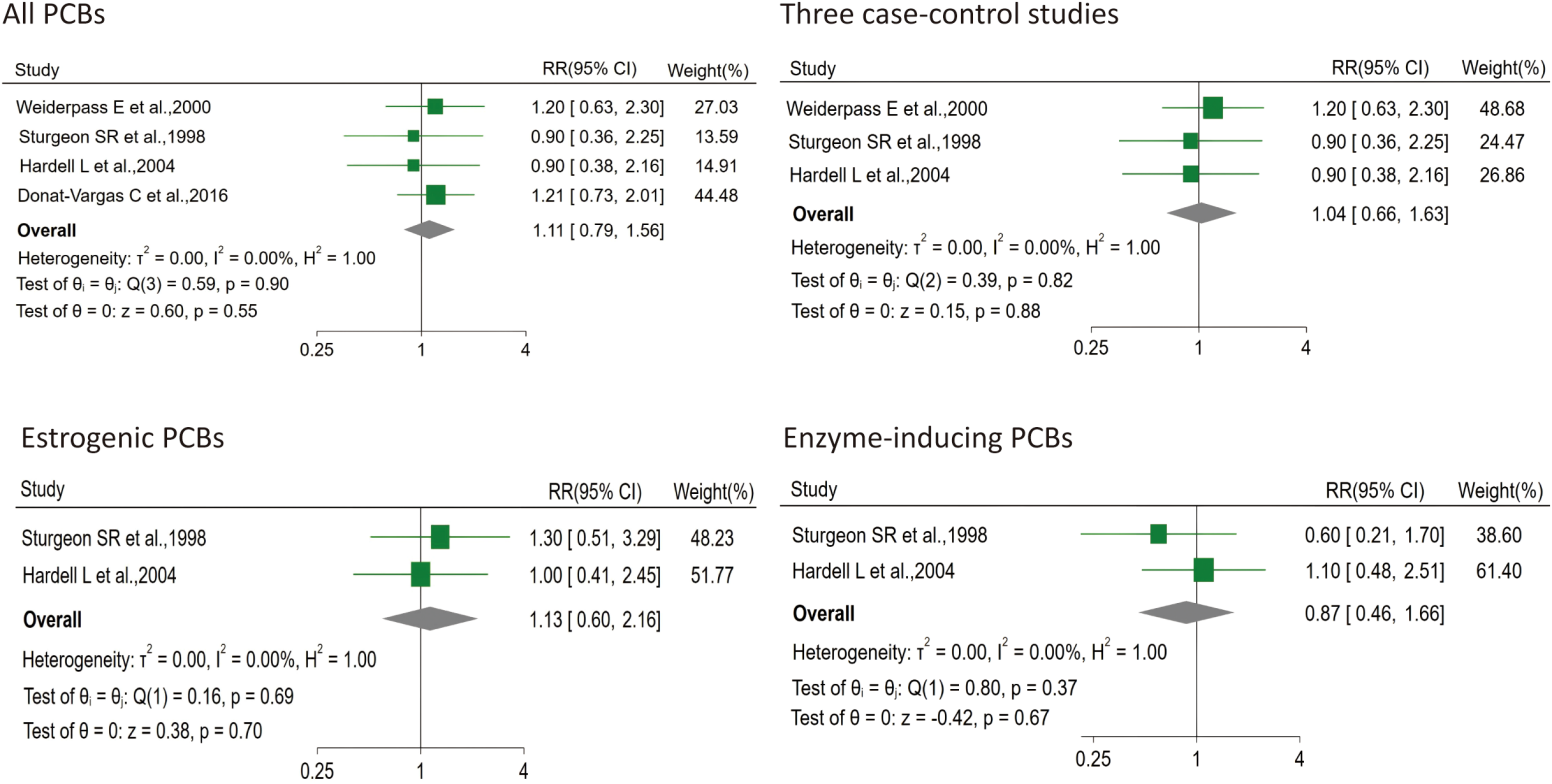
Forest plot for the association between PCB exposure and endometrial cancer risk.

In the subgroup analysis restricted to three case-control studies, the pooled estimate likewise showed no significant association (RR = 1.04, 95% CI: 0.66-1.63). No heterogeneity was detected among these studies (I² = 0.0%, p = 0.88). The pooled effect obtained using the fixed-effects model was consistent with the result from the random-effects model. Further analysis according to the functional classification of PCBs showed no significant association between estrogenic PCB exposure and endometrial cancer risk (RR = 1.13, 95% CI: 0.60-2.16). Similarly, the pooled analysis of enzyme-inducing PCBs did not reveal a significant association (RR = 0.87, 95% CI: 0.46-1.66). All results are presented in **Figure 2**.

### 3.4 Association of cadmium exposure with endometrial cancer risk

Among the six included studies, the pooled analysis using a random-effects model indicated that higher cadmium exposure was associated with an increased risk of endometrial cancer (RR = 1.22, 95% CI: 1.01-1.47). Substantial heterogeneity was observed among the studies (I²= 72.17%, p = 0.04).

**Figure 3:**
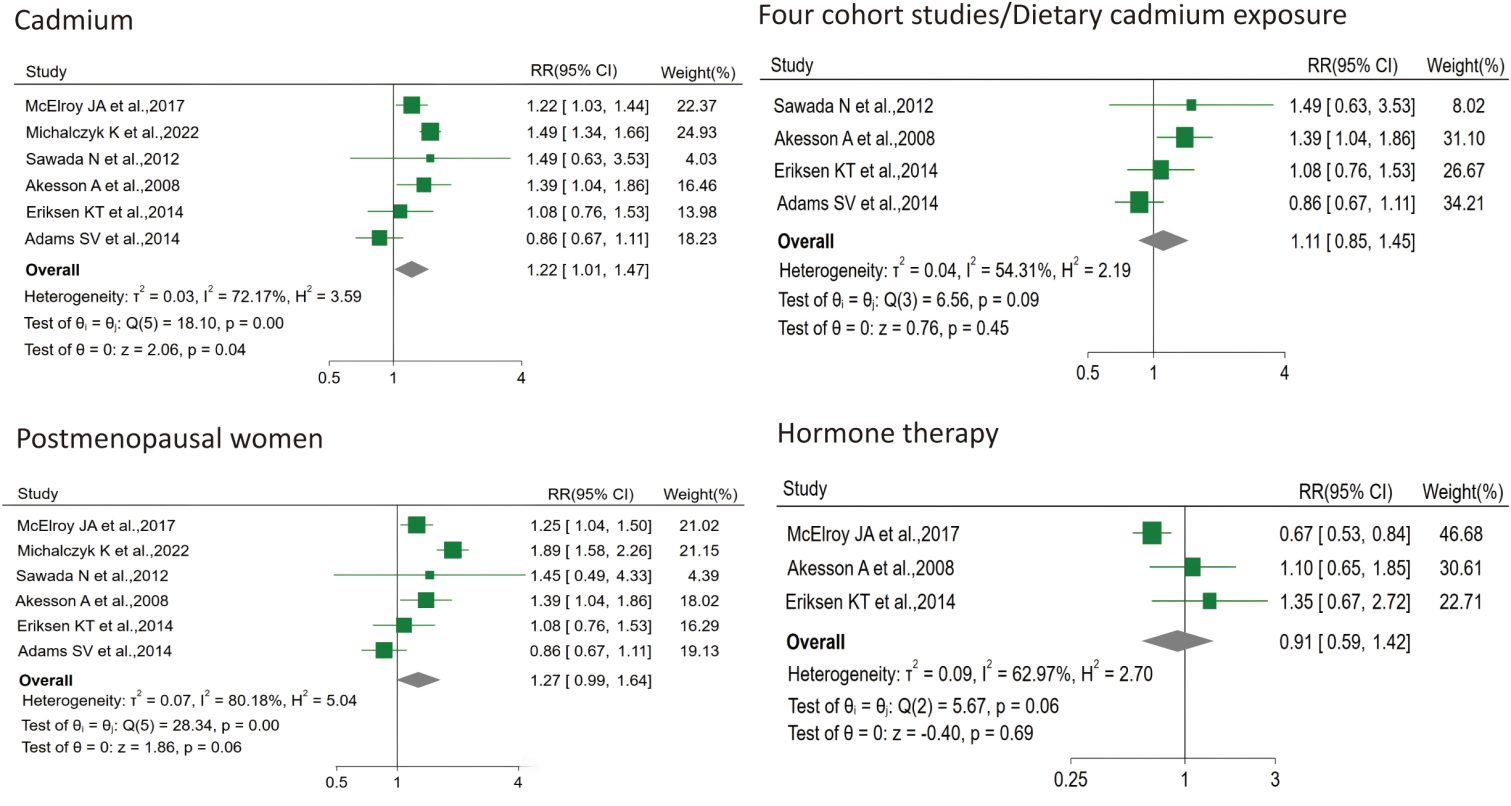
Forest plot for the association between cadmium exposure and endometrial cancer risk.

In four cohort studies restricted to dietary cadmium exposure, the pooled estimate showed no significant association (RR = 1.11, 95% CI: 0.85-1.45). Moderate heterogeneity was observed among these studies (I²= 54.31%, p = 0.45). Among postmenopausal women, cadmium exposure showed a borderline positive association with endometrial cancer risk (RR = 1.27, 95% CI: 0.99-1.64). In contrast, no significant association between cadmium exposure and endometrial cancer risk was observed among women receiving hormone therapy (RR = 0.91, 95% CI: 0.59-1.42). Moderate to substantial heterogeneity was observed across these subgroup analyses. All results are presented in **Figure 3**.

### 3.5 Sensitivity analyses

**Figure 4:**
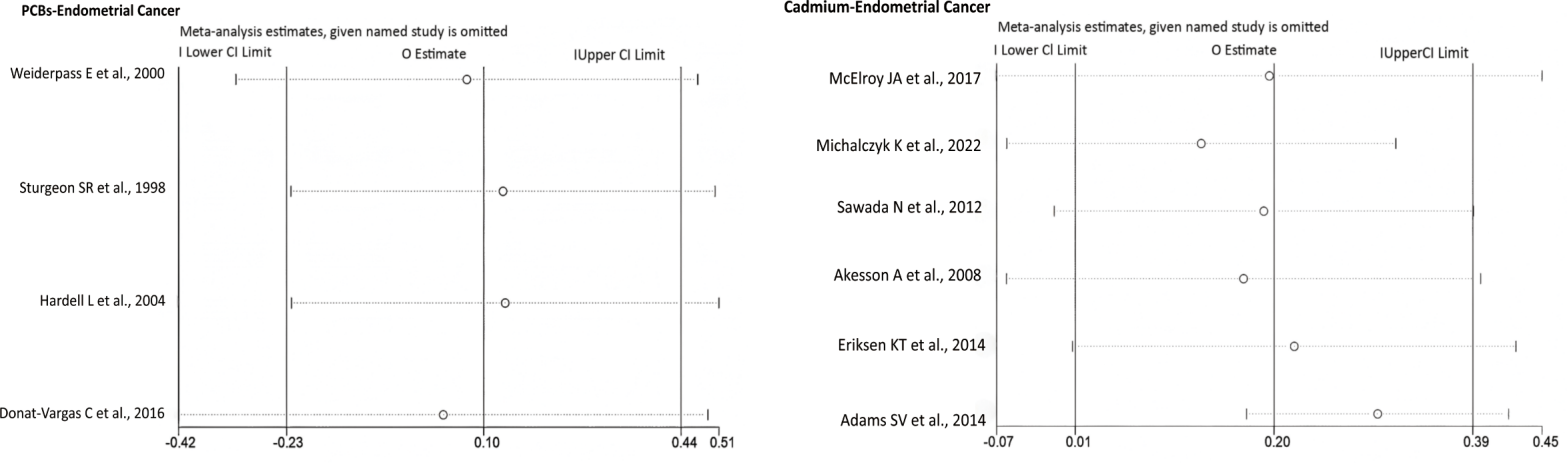
Sensitivity analysis of the associations between PCBs, cadmium, and endometrial cancer.

Sensitivity analysis was performed using a leave-one-out method to assess the robustness of the results. In the analysis of PCB exposure, the overall direction of the pooled effect remained consistent after excluding each study in turn. Although the effect estimates and their 95% confidence intervals showed some variation, the study by Donat-Vargas C was close to or crossed the null line. However, no single study was found to have a decisive influence on the overall results. The sensitivity analysis for cadmium exposure showed that the pooled effect estimates changed only slightly. The direction of the effect remained stable, and the 95% confidence intervals did not show substantial deviation (**Figure 4**).

### 3.6 Narrative evidence for PFAS, phthalates, and bisphenols

Studies examining the associations of PFAS, phthalates, and bisphenols with endometrial cancer were summarized using a narrative review approach (**Supplementary File 3**). Current evidence remains insufficient to establish a clear relationship between these three classes of compounds and the risk of endometrial cancer. Future prospective studies with larger populations, incorporating repeated exposure measurements and mechanistic investigations, are needed to further clarify their potential roles.

#### 3.6.1 Narrative evidence on PFAS and endometrial cancer

Epidemiological evidence regarding the association between PFAS exposure and endometrial cancer risk remains extremely limited, with only one case-control study currently available (18). This study included 430 women with endometrial cancer and 448 healthy controls matched by age and study center. Individual exposure levels were assessed by measuring serum concentrations of several PFAS, including EtFOSAA, MeFOSAA, PFHxS, PFOS, PFOA, and PFNA. The findings did not indicate a clear association between serum PFAS levels and the risk of endometrial cancer. However, certain PFAS, such as EtFOSAA, showed a modest positive association in analyses using continuous variables and within subgroups of postmenopausal women and type I tumors, suggesting the potential presence of a nonlinear relationship. Although serum samples were collected before diagnosis, reducing the likelihood of reverse causation, the results should still be interpreted with caution. Limitations include reliance on a single exposure measurement, lack of assessment of mixed-exposure effects, uncertainty regarding nonlinear dose-response patterns, and limited generalizability because most participants were postmenopausal women from a single U.S. cohort.

#### 3.6.2 Narrative evidence on phthalates and endometrial cancer

A total of two case-control studies were included, both using urinary phthalate metabolites as indicators of exposure (10). In both studies, women with confirmed endometrial cancer were compared with healthy controls. One study employed a nested case-control design and measured multiple urinary phthalate metabolites using samples collected before diagnosis, thereby partially reducing the likelihood of reverse causation (19).

Regarding specific findings, the study by Lin et al. (10) reported that MBzP was the only metabolite independently associated with endometrial cancer risk. The study by Sarink et al. (19) indicated that certain metabolites, including MnBP and a combined measure of DBP metabolites, were associated with an increased risk of endometrial cancer within specific exposure quantiles. However, no clear dose-response relationship was identified, and the risk estimates were unstable. Both studies were limited by reliance on single urine measurements, potential exposure misclassification, residual confounding from lifestyle and consumer-product use, inconsistent dose-response relationships, limited statistical precision for individual metabolites, and the absence of systematic evaluation of mixed-exposure effects.

#### 3.6.3 Narrative evidence on bisphenols and endometrial cancer

Evidence regarding the association between bisphenol exposure and endometrial cancer risk remains limited and inconsistent. A pilot case-control study conducted in Italy (20) reported elevated BPA concentrations in the blood and urine of women with endometrial cancer, while lower BPA levels were observed in tumor tissues. These findings suggest that BPA may exert its effects through systemic endocrine disruption rather than local tissue accumulation. Although the study involved a relatively small sample size, the analysis of multiple biological matrices, including blood, urine, and tissue, provided preliminary insights into potential mechanisms. In contrast, a nested case-control study within a multiethnic cohort did not observe a significant association between urinary BPA levels and endometrial cancer risk (19). These discrepancies may be related to limited sample sizes, reliance on single exposure measurements, incomplete covariate adjustment, and uncertainty regarding whether BPA acts independently or as part of a broader chemical mixture.

## 4 Discussion

### 4.1 Overall evidence landscape of EDCs and endometrial cancer

**Figure 5:**
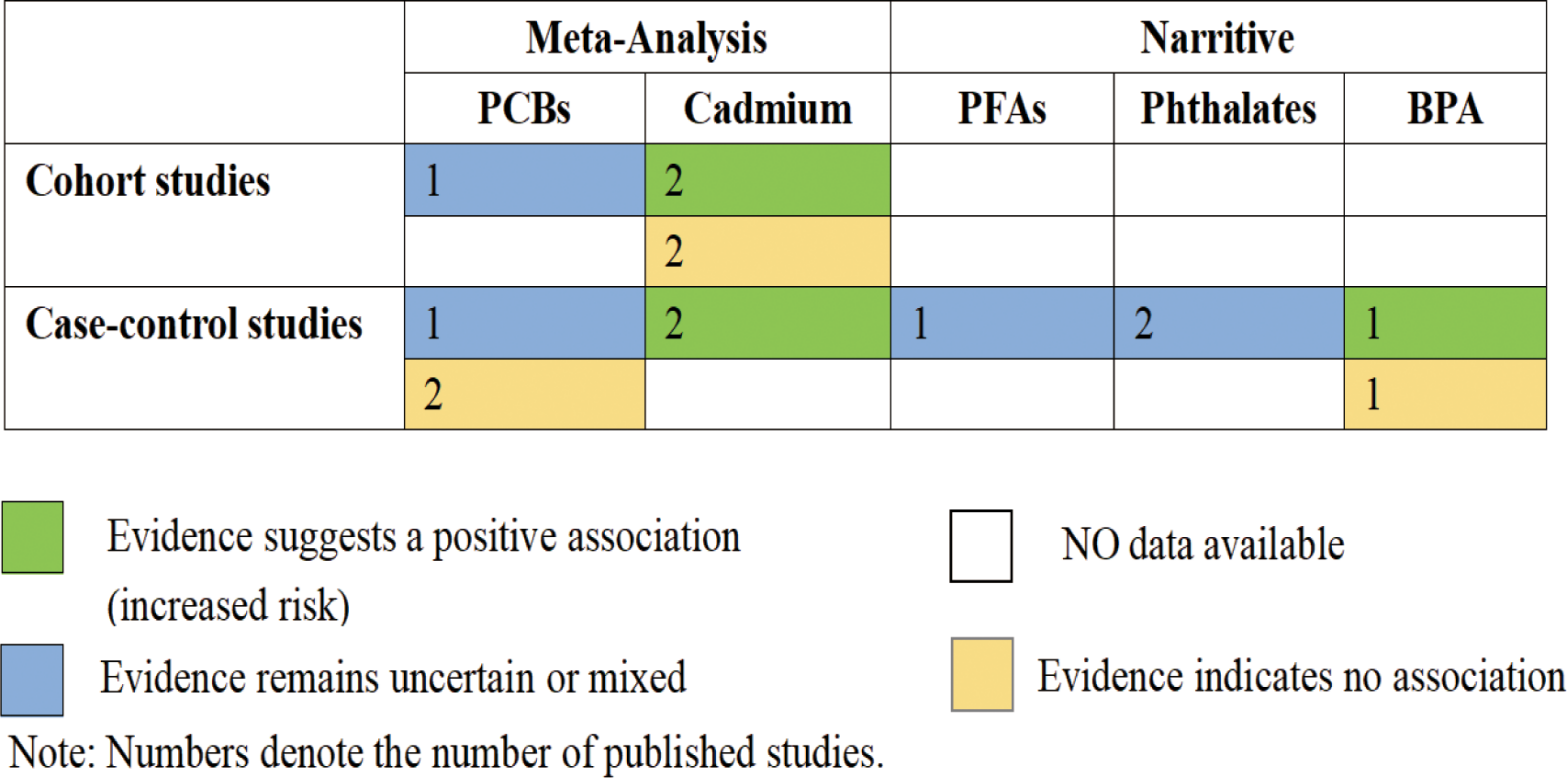
Evidence map of the associations between all endocrine-disrupting chemicals and endometrial cancer. The table summarizes the number of studies included in meta-analyses and narrative reviews, stratified by study design (cohort or case-control) and type of chemical exposure. Numbers in each cell denote the number of published studies for each chemical in the corresponding study type. Color coding indicates the direction and strength of evidence: green, evidence suggests a positive association (increased risk); blue, evidence remains uncertain or mixed; yellow, evidence indicates no association.

To visually summarize the distribution of evidence, study design composition, and overall direction of associations for different EDCs, an evidence map was constructed (**Figure 5**). This map provides an overview of the current evidence structure available for both quantitative synthesis and narrative analysis.

### 4.2 Interpretation of the association between PCBs exposure and endometrial cancer risk

The meta-analysis conducted in this study did not identify a statistically significant association between PCBs exposure and the risk of endometrial cancer. This pattern remained consistent across analyses of overall PCBs exposure, as well as subgroup analyses based on functional categories, including estrogenic PCBs and enzyme-inducing PCBs. Between-study heterogeneity was minimal. Similar results were obtained using both random-effects and fixed-effects models. In sensitivity analyses, sequential exclusion of individual studies did not materially change the direction of the pooled effect estimates. These findings indicate that the overall results are relatively robust within the context of the currently available evidence. As typical persistent organic pollutants, PCBs have long been a concern due to their potential toxic effects on the female reproductive system (30). Experimental evidence suggests that certain PCB congeners can interact with hormone receptors, disrupt endocrine pathways (31), or modify estrogen metabolism by inducing cytochrome P450 enzymes (32). However, epidemiological evidence indicates that these biological effects may not necessarily translate into a measurable increase in endometrial cancer risk at environmental exposure levels commonly observed in human populations (5).

The biological activities and modes of action differ substantially among PCB congeners *in vivo*. Some congeners exhibit estrogenic activity, whereas others show anti-estrogenic effects or primarily act through enzyme induction pathways (33). Such heterogeneity in biological effects may offset each other in analyses of overall exposure, resulting in pooled risk estimates close to the null. Overall, current evidence does not support a clear association between PCBs exposure and endometrial cancer risk. However, caution is warranted in interpreting these results because the observed null associations may be influenced by (1) variability in congener-specific biological activity potentially diluting observable effects, (2) generally low environmental exposure levels among study populations, (3) unmeasured confounding factors such as BMI, diet, and occupational exposures, and (4) limitations inherent to observational study designs, which preclude definitive causal inference. These considerations suggest that the findings should be interpreted as exploratory rather than conclusive.

### 4.3 Interpretation of the association between cadmium exposure and endometrial cancer risk

The meta-analysis suggested an overall positive association between cadmium exposure and the risk of endometrial cancer. Pooled estimates indicated that higher levels of cadmium exposure were generally associated with an increased risk of endometrial cancer. Sensitivity analyses showed that sequential removal of individual studies resulted in only minor changes in the pooled effect estimates and their 95% confidence intervals. The direction of the association remained largely consistent, and no individual study appeared to substantially influence on the overall results. These observations suggest that the observed association may be reasonably stable, though caution is warranted.

Cadmium is considered a metal with “estrogen-like” properties. It may theoretically promote the development of estrogen-related tumors by activating estrogen receptors, regulating the transcription of related genes (34), and interfering with hormone metabolism (35, 36). Caution is warranted in interpreting these results because differences in exposure assessment methods, population characteristics, and potential unmeasured confounding factors may influence the observed associations. Considerable variability exists in the methods used to assess cadmium exposure across epidemiological studies, including dietary intake estimated from food frequency questionnaires (37) and measurements of cadmium concentrations in blood or urine (38, 39), which reflect different exposure windows and are not directly comparable. In the present study, subgroup analyses restricted to cohort studies using dietary cadmium as the exposure source showed reduced heterogeneity between studies. This finding may reflect greater consistency in study design and exposure assessment rather than a more accurate estimation of exposure. Dietary cadmium generally reflects long-term, low-dose chronic exposure (40), but is also subject to measurement error, which may attenuate risk estimates toward the null and reduce variability across studies. In contrast, biomarker measurements may better capture internal exposure levels but can also be influenced by recent exposure, inter-individual metabolic differences, and renal function status (41), thereby contributing to increased between-study heterogeneity.

Among postmenopausal women, this study observed a borderline positive association between cadmium exposure and endometrial cancer risk, a trend that has also been reported in previous research (28). In postmenopausal women, estrogen is primarily derived from peripheral tissue conversion. Under conditions of relatively low endogenous estrogen levels, environmental chemicals with estrogen-like activity may exert more pronounced relative biological effects (42). In contrast, no significant association was observed among women using hormone therapy. This finding is consistent with analyses from the Women’s Health Initiative study (25). These findings suggest that the influence of cadmium may be modified by exogenous hormone exposure, population characteristics, and exposure measurement, and that the observed associations should be interpreted cautiously as exploratory. Future prospective studies with precise exposure assessment and rigorous designs are needed to clarify these associations (43).

### 4.4 PFAS, phthalates, and bisphenols: an emerging evidence landscape

Current epidemiological evidence linking PFAS, phthalates, and bisphenols with endometrial cancer remains at an exploratory stage (3, 11, 44, 45). Most studies rely on single measurements of blood or urinary biomarkers, which may not adequately capture long-term cumulative exposure (46, 47). Moreover, evidence on the health effects of chronic low-dose exposure remains limited. These chemicals typically occur as mixtures in the environment and may produce synergistic or antagonistic effects. However, most existing studies focus on individual compounds and lack a systematic evaluation of mixed exposures and their potential interactions. Mechanistic studies indicate that PFAS, phthalates, and bisphenols can disrupt estrogen-related gene expression, influence proliferation and apoptosis of endometrial cells, and induce histological alterations in animal or *in vitro* models (48–50). These findings provide biological plausibility for potential carcinogenic effects. Nevertheless, due to the limited epidemiological evidence in human populations and the instability of current risk estimates, a clear causal inference cannot yet be established. Although signals suggesting possible associations with endometrial cancer have been reported, further well-designed prospective studies are needed to clarify their long-term health risks.

### 4.5 Mechanistic integration of EDCs in Endometrial cancer

**Figure 6:**
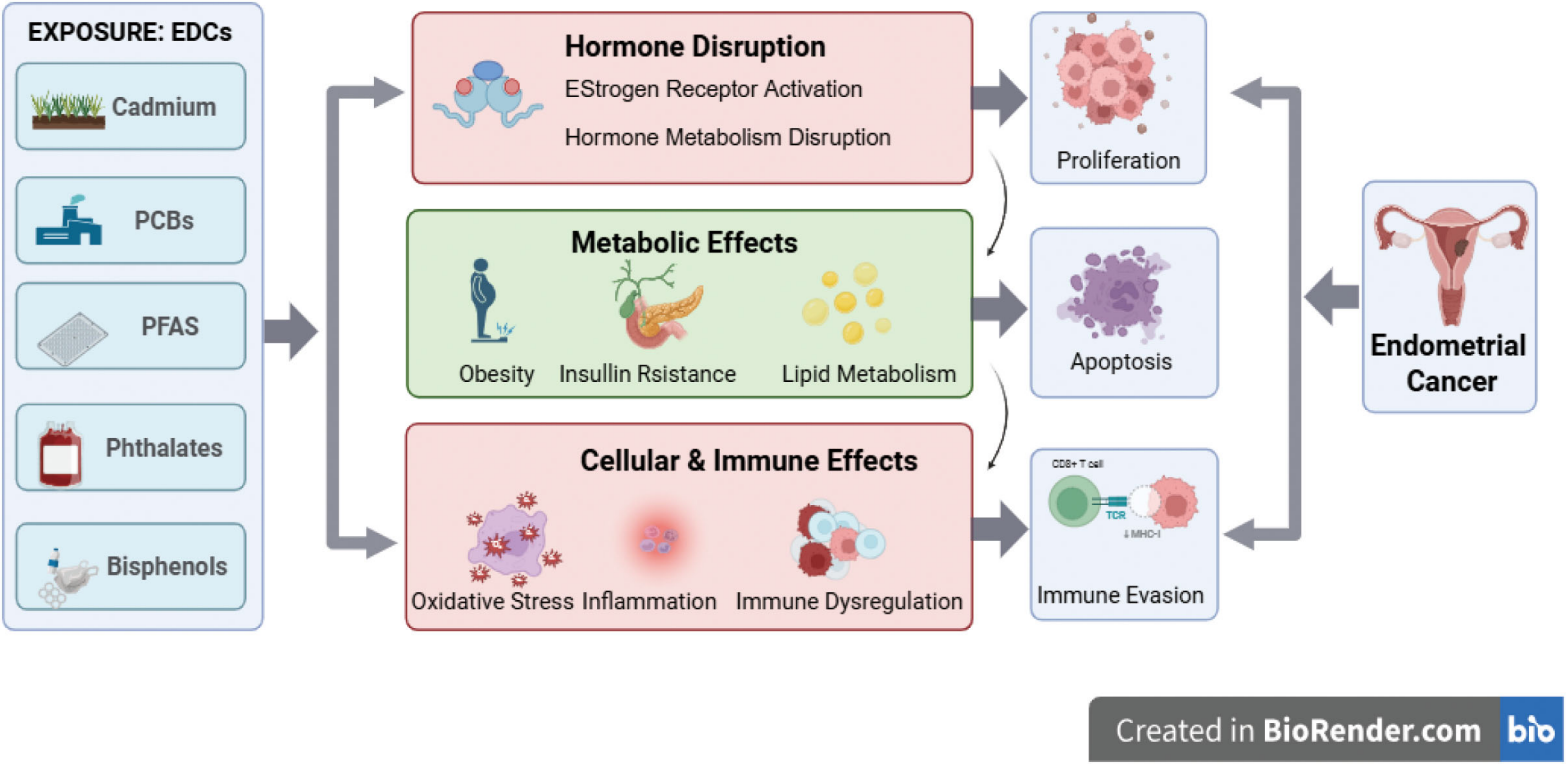
Proposed mechanistic pathways linking EDCs to endometrial cancer. This schematic illustrates the potential pathways through which EDCs may contribute to endometrial cancer development. EDCs included in this diagram are cadmium, PCBs, PFAS, phthalates, and BPA. Exposure to EDCs can lead to (1) hormone disruption, including estrogen receptor activation and hormone metabolism disruption; (2) metabolic effects, including obesity, insulin resistance, and altered lipid metabolism; and (3) cellular and immune effects, including oxidative stress, inflammation, and immune dysregulation. These pathways may induce proliferation, apoptosis dysregulation, and immune evasion, ultimately contributing to endometrial carcinogenesis. Arrows indicate the direction of effect. Figure icons represent key cellular or metabolic processes. (Figure was created using https://app.biorender.com/).

Although EDCs differ in chemical structure and exposure patterns, they may exert convergent effects by acting on shared biological pathways involved in endometrial carcinogenesis. A key mechanism involves disruption of estrogen signaling, whereby EDCs can directly activate estrogen receptors or interfere with hormone synthesis and metabolism, leading to sustained endometrial proliferation (51, 52). In addition, EDCs exposure has been associated with metabolic disturbances, including obesity and insulin resistance (53), as well as enhanced oxidative stress and chronic inflammation (54), all of which are recognized contributors to tumor initiation and progression. Emerging evidence further suggests that EDCs may promote carcinogenesis by modulating the tumor immune microenvironment (55, 56). Taken together, these interconnected biological pathways provide a plausible mechanistic basis linking EDCs exposure to an increased risk of endometrial cancer (**Figure 6**).

## 5 Limitations

Although meta-analyses were conducted for cadmium and PCBs, the number of eligible studies was limited. Moreover, substantial heterogeneity was observed in the cadmium-related analyses, which may be attributable to differences in exposure assessment methods, population characteristics, and control of confounding factors. In addition, studies examining PFAS, phthalates, and bisphenols were relatively scarce. Most relied on single biological sample measurements, which may not accurately reflect long-term or cumulative exposure levels. Furthermore, systematic evaluation of mixed exposure effects from multiple endocrine-disrupting chemicals remains lacking. Some included studies were primarily case-control in design. Consequently, residual confounding and selection bias cannot be fully excluded, which may limit the strength of causal inference. Several limitations should be acknowledged. First, most included studies were observational, and residual confounding by lifestyle and environmental factors cannot be excluded. Second, exposure assessment varied across studies, including single-time biomarker measurements and dietary questionnaires, which may not accurately capture long-term or cumulative exposure. Third, the number of studies for emerging EDCs (PFAS, phthalates, bisphenols) was limited, and most were case-control studies, which may introduce selection bias. Finally, the findings may have limited generalizability and do not establish causality.

## 6 Conclusion

This systematic review and meta-analysis indicate that the associations between different environmental endocrine-disrupting chemicals and endometrial cancer risk vary substantially. Cadmium exposure showed an epidemiological signal suggestive of an increased risk of endometrial cancer. However, this association was not consistent across different exposure sources and population subgroups. In contrast, current evidence does not support a significant association between PCBs exposure and endometrial cancer risk. This consistently null finding helps clarify inconsistencies reported in previous studies. For emerging endocrine-disrupting chemicals including PFAS, phthalates, and bisphenols, the available evidence remains limited. Further well-designed prospective studies are required to verify their potential roles in endometrial cancer development. From a clinical and public health perspective, these findings highlight environmental endocrine disruptors as potentially modifiable risk factors for endometrial cancer. Although current evidence does not support specific screening or therapeutic recommendations, reducing exposure to these chemicals may represent a complementary strategy for endometrial cancer prevention, particularly among women with established hormonal and metabolic risk factors.

## Data Availability

The original contributions presented in the study are included in the article/**Supplementary Material**. Further inquiries can be directed to the corresponding author.
