# Supplementary material for: Environmental endocrine disruptors and endometrial cancer risk: a systematic review and meta-analysis of cadmium and polychlorinated biphenyls, with emerging evidence on PFAS, phthalates, and bisphenols
Source: Front Oncol. 2026 May 28;16:1848363. doi: 10.3389/fonc.2026.1848363 (PMC13253379; doi:10.3389/fonc.2026.1848363)
Supplement: Supplementary file 1 [file Table1.doc]

**Supplementary File 1**

1. **Search strategy for MEDLINE (PubMed) 926**

#1 ("endocrine disrupt*"[Title/Abstract] OR "endocrine disruptor*"[Title/Abstract]

OR "endocrine disrupting chemical*"[Title/Abstract]

OR "endocrine disrupting effect*"[Title/Abstract]

OR EDC[Title/Abstract] OR EDCs[Title/Abstract]

OR "environmental pollutant*"[Title/Abstract]

OR "environmental chemical*"[Title/Abstract])

#2 ("polychlorinated biphenyl*"[Title/Abstract]

OR PCB[Title/Abstract] OR PCBs[Title/Abstract]

OR "polychlorinated biphenyls"[MeSH Terms])

#3 ("perfluoroalkyl"[Title/Abstract] OR "polyfluoroalkyl"[Title/Abstract]

OR perfluoro*[Title/Abstract] OR polyfluoro*[Title/Abstract]

OR PFAS[Title/Abstract] OR PFOA[Title/Abstract]

OR PFOS[Title/Abstract] OR PFHxS[Title/Abstract]

OR PFNA[Title/Abstract])

#4 (phthalate*[Title/Abstract]

OR "di(2-ethylhexyl) phthalate"[Title/Abstract]

OR DEHP[Title/Abstract] OR DnBP[Title/Abstract]

OR DEP[Title/Abstract] OR MEHP[Title/Abstract]

OR MEOHP[Title/Abstract] OR MEHHP[Title/Abstract])

#5 (bisphenol*[Title/Abstract] OR BPA[Title/Abstract]

OR BPS[Title/Abstract] OR BPF[Title/Abstract])

#6 (cadmium[Title/Abstract] OR "cadmium compounds"[MeSH Terms]

OR lead[Title/Abstract] OR arsenic[Title/Abstract]

OR mercury[Title/Abstract] OR chromium[Title/Abstract]

OR nickel[Title/Abstract] OR "heavy metal"[Title/Abstract]

OR "heavy metals"[Title/Abstract] OR "toxic metal"[Title/Abstract])

#7 #1 OR #2 OR #3 OR #4 OR #5 OR #6

#8 ("endometrial cancer"[Title/Abstract] OR "endometrial carcinoma"[Title/Abstract]

OR "endometrial neoplasm"[Title/Abstract]

OR "uterine cancer"[Title/Abstract]

OR "uterine carcinoma"[Title/Abstract]

OR "uterine neoplasm"[Title/Abstract]

OR "corpus uteri neoplasms"[MeSH Terms])

#9 #7 AND #8

The last search was run on November 30, 2025.

1. **Search strategy for Embase (Ovid) 167**

#1 endocrine disrupt*.ti,ab.

OR endocrine disruptor*.ti,ab.

OR "endocrine disrupting chemical*".ti,ab.

OR EDC.ti,ab. OR EDCs.ti,ab.

OR "environmental pollutant*".ti,ab.

OR "environmental chemical*".ti,ab.

#2 "polychlorinated biphenyl*".ti,ab.

OR PCB.ti,ab. OR PCBs.ti,ab.

OR 'polychlorinated biphenyl'/exp

#3 perfluoroalkyl.ti,ab. OR polyfluoroalkyl.ti,ab.

OR perfluoro*.ti,ab. OR polyfluoro*.ti,ab.

OR PFAS.ti,ab. OR PFOA.ti,ab.

OR PFOS.ti,ab. OR PFHxS.ti,ab. OR PFNA.ti,ab.

#4 phthalate*.ti,ab.

OR "di(2-ethylhexyl) phthalate".ti,ab.

OR DEHP.ti,ab. OR DnBP.ti,ab.

OR DEP.ti,ab. OR MEHP.ti,ab.

OR MEOHP.ti,ab. OR MEHHP.ti,ab.

#5 bisphenol*.ti,ab. OR BPA.ti,ab.

OR BPS.ti,ab. OR BPF.ti,ab.

#6 cadmium.ti,ab. OR 'cadmium compound'/exp

OR lead.ti,ab. OR arsenic.ti,ab.

OR mercury.ti,ab. OR chromium.ti,ab.

OR nickel.ti,ab.

OR "heavy metal".ti,ab. OR "heavy metals".ti,ab.

OR "toxic metal".ti,ab.

#7 #1 OR #2 OR #3 OR #4 OR #5 OR #6

#8 "endometrial cancer".ti,ab.

OR "endometrial carcinoma".ti,ab.

OR "endometrial neoplasm".ti,ab.

OR "uterine cancer".ti,ab.

OR "uterine carcinoma".ti,ab.

OR "uterine neoplasm".ti,ab.

OR 'corpus uteri tumor'/exp

#9 #7 AND #8

1. **Search strategy for Web of Science 3178**

#1 TS=("endocrine disrupt*" OR "endocrine disruptor*"

OR "endocrine disrupting chemical*"

OR EDC OR EDCs

OR "environmental pollutant*"

OR "environmental chemical*")

#2 TS=("polychlorinated biphenyl*" OR PCB OR PCBs)

#3 TS=(perfluoroalkyl OR polyfluoroalkyl OR perfluoro*

OR PFOS OR PFOA OR PFHxS OR PFNA OR PFAS)

#4 TS=(phthalate* OR "di(2-ethylhexyl) phthalate"

OR DEHP OR DnBP OR DEP OR MEHP OR MEOHP OR MEHHP)

#5 TS=(bisphenol* OR BPA OR BPS OR BPF)

#6 TS=(cadmium OR lead OR arsenic OR mercury OR chromium

OR nickel OR "heavy metal" OR "heavy metals" OR "toxic metal")

#7 #1 OR #2 OR #3 OR #4 OR #5 OR #6

#8 TS=("endometrial cancer" OR "endometrial carcinoma"

OR "endometrial neoplasm" OR "uterine cancer"

OR "uterine carcinoma" OR "uterine neoplasm"

OR "corpus uteri neoplasm")

#9 #7 AND #8

**4.Search strategy for Cochrane Library 2629**

#1(endocrine disrupt*:ti,ab

OR endocrine disruptor*:ti,ab

OR endocrine disrupting chemical*:ti,ab

OR EDC*:ti,ab

OR environmental pollutant*:ti,ab

OR environmental chemical*:ti,ab)

#2 ("polychlorinated biphenyl*":ti,ab OR PCB:ti,ab OR PCBs:ti,ab

OR [mh "polychlorinated biphenyls"])

#3 (perfluoroalkyl:ti,ab OR polyfluoroalkyl:ti,ab

OR perfluoro*:ti,ab OR PFAS:ti,ab

OR PFOS:ti,ab OR PFOA:ti,ab

OR PFHxS:ti,ab OR PFNA:ti,ab)

#4 (phthalate*:ti,ab OR "di(2-ethylhexyl) phthalate":ti,ab

OR DEHP:ti,ab OR DnBP:ti,ab

OR DEP:ti,ab OR MEHP:ti,ab

OR MEOHP:ti,ab OR MEHHP:ti,ab)

#5 (bisphenol*:ti,ab OR BPA:ti,ab

OR BPS:ti,ab OR BPF:ti,ab)

#6 (cadmium:ti,ab OR [mh "cadmium compounds"]

OR lead:ti,ab OR arsenic:ti,ab

OR mercury:ti,ab OR chromium:ti,ab

OR nickel:ti,ab OR "heavy metal":ti,ab

OR "heavy metals":ti,ab OR "toxic metal":ti,ab)

#7 #1 OR #2 OR #3 OR #4 OR #5 OR #6

#8 ("endometrial cancer":ti,ab OR "endometrial carcinoma":ti,ab

OR "endometrial neoplasm":ti,ab

OR "uterine cancer":ti,ab OR "uterine carcinoma":ti,ab

OR "uterine neoplasm":ti,ab

OR [mh "corpus uteri neoplasms"])

#9 #7 AND #8
