# Supplementary material for: Environmental endocrine disruptors and endometrial cancer risk: a systematic review and meta-analysis of cadmium and polychlorinated biphenyls, with emerging evidence on PFAS, phthalates, and bisphenols
Source: Front Oncol. 2026 May 28;16:1848363. doi: 10.3389/fonc.2026.1848363 (PMC13253379; doi:10.3389/fonc.2026.1848363)
Supplement: Supplementary file 2 [file Table2.doc]

**Environmental endocrine disruptors and endometrial cancer risk: a systematic review and meta-analysis of cadmium and polychlorinated biphenyls, with emerging evidence on PFAS, phthalates, and bisphenols**

Lutian Gong1 , Wenping Lu*1, Yujing Zhao1, Heting Mei1, 2, Xiangyang Zhang1, 2

1 Department of Oncology, China Academy of Chinese Medical Sciences Guang’anmen Hospital,

Beijing, China

2 Beijing University of Chinese Medicine, Beijing, China

*CORRESPONDENCE

Wenping Lu

lu_wenping@sina.com

**Supplementary File 2**

**Table 1** Quality assessment of the Case-control study

| **Study design** | **Study** | **Selection** | | | | **Comparability** | | **Exposure** | | | **Quality score** |
| --- | --- | --- | --- | --- | --- | --- | --- | --- | --- | --- | --- |
|  |  | **Item 1** | **Item 2** | **Item 3** | **Item4** | **Item 5a** | **Item 5b** | **Item 6** | **Item7** | **Item 8** |  |
| Case-control study | Madrigal et al., 2025 | 1 | 1 | 1 | 1 | 1 | 1 | 1 | 1 | 1 | 9 |
|  | Lin et al., 2025 | 1 | 1 | 1 | 1 | 1 | 1 | 1 | 1 | 0 | 8 |
|  | Sarink et al., 2021 | 1 | 1 | 1 | 1 | 1 | 1 | 1 | 1 | 1 | 9 |
|  | Aquino et al., 2019 | 1 | 0 | 0 | 1 | 0 | 0 | 1 | 1 | 0 | 4 |
|  | Weiderpass et al., 2000 | 1 | 1 | 1 | 1 | 1 | 1 | 1 | 1 | 1 | 9 |
|  | Sturgeon et al., 1998 | 1 | 1 | 1 | 1 | 1 | 1 | 1 | 1 | 1 | 9 |
|  | Hardell et al., 2004 | 1 | 1 | 1 | 0 | 0 | 1 | 1 | 1 | 0 | 6 |
|  | McElroy et al., 2017 | 1 | 1 | 1 | 1 | 1 | 1 | 1 | 1 | 1 | 9 |
|  | Michalczyk et al.,2022 | 1 | 1 | 1 | 1 | 1 | 0 | 1 | 1 | 0 | 7 |

**Table 2** Quality assessment of the Cohort study

| **Study design** | **Study** | **Selection** | | | | **Comparability** | | **Outcome** | | | **Quality score** |
| --- | --- | --- | --- | --- | --- | --- | --- | --- | --- | --- | --- |
|  |  | **Item 1** | **Item 2** | **Item 3** | **Item4** | **Item 5a** | **Item 5b** | **Item 6** | **Item7** | **Item 8** |  |
| Cohort study | Donat-Vargas et al., 2016 | 1 | 1 | 1 | 1 | 1 | 1 | 1 | 1 | 1 | 9 |
|  | Sawada et al., 2012 | 1 | 1 | 1 | 1 | 1 | 1 | 1 | 1 | 1 | 9 |
|  | Akesson et al., 2008 | 1 | 1 | 1 | 1 | 1 | 1 | 1 | 1 | 1 | 9 |
|  | Eriksen et al., 2014 | 1 | 1 | 1 | 1 | 1 | 1 | 1 | 1 | 1 | 9 |
|  | Adams et al., 2014 | 1 | 1 | 1 | 1 | 1 | 1 | 1 | 1 | 1 | 9 |

The study quality was assessed according to the Newcastle Ottawa Quality assessment scale. This scale awards a maximum of 9 points to each study, 1 = “Yes”, 0 = “No”, “Unable to determine” or “Not available”.

For case-control study: Item 1: Is the case definition adequate; Item 2: Representativeness of the cases; Item 3: Selection of Controls; Item 4: Definition of Controls; Item 5a: Study controls for (Select the most important factor); Item 5b: Study controls for any additional factor; Item 6: Ascertainment of exposure; Item 7: Same method of ascertainment for cases and controls; Item 8: Non-Response rate.

For cohort study, Item 1: Representativeness of the exposed cohort; Item 2: Selection of the non-exposed cohort; Item 3: Ascertainment of exposure; Item 4: Demonstration that outcome of interest was not present at start of study; Item 5a: study controls for (select the most important factor);Item 5b: study controls for any additional factor; Item 6: Assessment of outcome; Item 7: Was follow-up long enough for outcomes to occur; Item 8: Adequacy of follow up of cohorts.
