# Supplementary material for: Environmental endocrine disruptors and endometrial cancer risk: a systematic review and meta-analysis of cadmium and polychlorinated biphenyls, with emerging evidence on PFAS, phthalates, and bisphenols
Source: Front Oncol. 2026 May 28;16:1848363. doi: 10.3389/fonc.2026.1848363 (PMC13253379; doi:10.3389/fonc.2026.1848363)
Supplement: Supplementary file 3 [file Table3.doc]

**Supplementary File 3**

**Table 1**

Structured narrative summary of epidemiological evidence for per-and polyfluoroalkyl substances (PFAS), phthalates, and bisphenols in relation to endometrial cancer.

| **Exposure** | **Number of studies** | **Author,year** | **Study design** | **Sample Tested** | **Number of measurements** | **Whether it reflects long-term exposure** | | **Whether**  **mixed**  **exposure**  **was consi**  **dered** | | --- | | | **Main direction of**  **results** | | --- |  | |  | | --- |  |  | | --- | | | --- | --- | --- | | **Consistency of results** | **Key limitations** | **strength of evidence assessment** | **main sources of risk of bias** |
| --- | --- | --- | --- | --- | --- | --- | --- | --- | --- | --- | --- | --- |
| **PFAs** | 1 | Madrigal et al., 2025 | Case-control | serum | Single measurement | Partially reflects | Yes | | No association was | | --- |   observed between PFAS exposure and endometrial cancer. | Not assessable | Single measurement; limited age range of the study population | Evidence is insufficient but suggestive | Exposure misclassification bias; selection bias; residual confounding; time-window bias; detection limit handling bias |
| Phthalates | 2 | Lin et al.,2025 | Case-control | Urine | Single measurement | No | Partially considered | Some phthalate metabolites (MBzP) were associated with endometrial cancer. | inconsistent | Single biological sample; short-term exposure indicator; mixed exposures not comprehensively assessed | Limited and inconsistent | Reverse causation bias; selection bias; exposure misclassification bias; residual confounding |
|  |  | Sarink  et al.,2021 | Case-control | Urine | Single measurement | No | Partially considered | Some phthalate metabolites (MnBP) were associated with endometrial cancer. | inconsistent | Single measurement; small sample size; study population limited to postmenopausal women; mixed exposures not comprehensively assessed | Limited and inconsistent | Exposure misclassification bias; residual confounding; detection limit handling bias |
| Bisphenols | 2 | Aquino et al.,2019 | Case-control | blood, urine ,Endometrium tissue sample | Single measurement | No | No | Bisphenol A (BPA) levels in urine and blood were partially associated with endometrial cancer. | inconsistent | Small sample size; short-term exposure indicator; single measurement; mixed exposures not considered | Limited and inconsistent | Small sample size; reverse causation bias; selection bias; confounding bias; single time-point measurement |

**Note:** Sarink et al., 2021 investigated both BPA and phthalates
